# The diagnostic and prognostic values of microRNA-196a in cancer

**DOI:** 10.1042/BSR20203559

**Published:** 2021-01-07

**Authors:** Mengqiu Xiong, Ping Wang, Bei Pan, Junjie Nie, Shukui Wang, Bangshun He

**Affiliations:** 1 General Clinical Research Center, Nanjing First Hospital, Nanjing Medical University, Nanjing 210006, China; 2 Department of Medical Biology, Wannan Medical College, Wuhu 241002,China; 3 Medical College, Southeast University, Nanjing 210006, China; 4 Collaborative Innovation Center For Cancer Personalized Medicine, Nanjing Medical University, Nanjing 210006, China

**Keywords:** cancer, diagnostics, microRNA

## Abstract

MicroRNA-196a (miR-196a) was previously reported to be up-regulated in cancers, and it has the diagnostic and prognostic values in cancers. Whereas, the conclusion was still unclear according to the published data. To assess such roles of miR-196a in cancers, the present study was conducted based on published data and online cancer-related databases. To identify the relevant published data, we searched articles in databases and then the relevant data were extracted to evaluate the correlation between miR-196a expression and diagnosis, prognosis for cancer patients. The pooled results showed that miR-196a was a valuable diagnostic biomarker in cancer (area under curve (AUC) = 0.87, 95% CI: 0.84–0.90; sensitivity (SEN) = 0.73, 95% CI: 0.64–0.81; specificity (SPE) = 0.90, 95% CI: 0.81–0.95), which was consistent with the data from databases (breast cancer: miR-196a-3p: AUC = 0.77, 95% CI: 0.74–0.79; miR-196a-5p: AUC = 0.71, 95% CI: 0.66–0.75; pancreatic cancer: miR-196a-3p: AUC = 0.80, 95% CI: 0.73–0.87; miR-196a-5p: AUC = 0.61, 95% CI: 0.51–0.71). In addition, the pooled result revealed that elevated miR-196a expression in tumor tissues (HR = 2.54, 95% CI: 1.79–3.61, *P_Heterogeneity_*=0.000, *I*^2^ = 75.8%) or serum/plasma (HR = 4.06, 95% CI: 2.67–6.18, *P_Heterogeneity_*=0.668, *I*^2^ = 0%) of patients was an unfavorable survival biomarker, which was consistent with the data from databases (adrenocortical carcinoma: HR = 5.70; esophageal carcinoma: HR = 1.93; brain lower grade glioma: HR = 2.91; GSE40267: HR = 2.47, 95% CI: 1.2–5.07; TCGA: HR = 1.82, 95% CI: 1.21–2.74; GSE19783: HR = 4.24, 95% CI: 1–18.06). In short, our results demonstrated that miR-196a in tumor tissue or serum/plasma could be used as a prognostic and diagnostic values for cancers.

## Background

MicroRNAs (miRNAs), a kind of non-coding RNAs with 21–25 nucleotides, inhibit gene expression by targeting the 3′-untranslated region (3′-UTR) of target messenger RNA (mRNA) [1]. In the past few decades, aberrant expression of miRNAs has been shown to play roles in tumorigenesis and tumor progression in a variety of cancers [2]. Meanwhile, studies of these molecules have led to the observation of clinically useful genetic biomarkers and novel therapeutic agents.

miR-196a, a member of the miR-196 family that has two members (miR-196a and miR-196b), comes from the transcription of two genomic loci, HOXC gene *MIR196A2* and HOXB gene *MIR196A1* [3]. miR-196a-5p and miR-196a-3p are two molecules produced by pre-MIR196A2. Moreover, pre-MIR196A1 also encodes miR-196a-5p. Previous studies have shown that miR-196a, acts as an oncogene, exert multiple functions in carcinogenesis and cancer progression, such as down-regulation of miR-196a inhibited proliferation and invasion of hepatocellular carcinoma (HCC) cells by targeting FOXO1 [4]; in breast cancer, overexpression of miR-196a promotes tumor growth and metastasis by targeting SPRED1 [5]; in osteosarcoma, it could promote cell migration, invasion and the epithelial–mesenchymal transition by targeting HOXA5 [6]. Whereas, in testicular germ cell tumor, it was also reported to repress cell proliferation, migration, invasion and tumor neurogenesis by inhibition of NR6A1/E-cadherin signaling axis [7]. Moreover, in head and neck cancer, cancer-associated fibroblasts derived exosomal miR-196a was responsible for cisplatin resistance by targeting CDKN1B and ING5 [8]. Meanwhile, polymorphism in miR-196a-2 was reported to confer occurrence risk or progression of cancers, such as it was associated with HCC recurrence after liver transplantation [9], and we also previously reported that it was associated with occurrence of cancers [10]. As a regulator, it could be also regulated by non-coding RNAs, such as lncRNA FEZF-AS1 [11], circRNA 101308 [12], H19 [13], lncRNA SNHG1 [14], which were involved in tumorigenesis and tumor progression.

Additionally, miR-196a was focused on cancers by studies for its biological function in carcinogenesis and potential role in cancer diagnosis or survival prediction. For patients with gliomas, elevated miR-196a expression was associated with aggressive pathological features and shorter survival [15]. Overexpression of miR-196a was reported in types of cancers, such as liver cancer [4,16], breast cancer [17], esophageal squamous cell carcinoma (ESCC) [18], thyroid carcinoma [19], esophageal carcinoma [20] etc. Moreover, the level of miR-196a-5p in serum was suggested to be served as a diagnostic biomarker for cancers, including non-small cell lung cancer (NSCLC) [21], prostate cancer [22] and biomarker of cancer metastasis [23]. Whereas, the conclusions of role of miR-196a in clinical application were not always consistent. Therefore, we conducted this meta-analysis according to published data and try to determine whether miR-196a is a valuable biomarker for cancer diagnosis and prognosis.

## Materials and methods

### Search strategy

In order to obtain all relevant articles, we used the keywords (‘microRNA-196a’ OR ‘miR-196a’ OR ‘microRNA-196a’) and (‘carcinoma’ OR ‘cancer’ OR ‘tumor’) to search in PubMed, Web of Science, CNKI database and other similar databases. In addition, we manually searched for related references in some additional papers and reviews. A total of 425 articles were searched from the three databases (PubMed, Web of Science and CNKI) by using the keywords and 98 duplicated articles were removed by screening the title, abstract and author and then the article type of reviews, letters or not related to the topic according to the established criteria were excluded. After reading full-text of 44 articles meeting the including criteria, 21 of them with insufficient data and unrelated to the diagnosis and prognosis were removed, and then a total of 23 studies were enrolled in the present study, see Supplementary Figure S1.

### Inclusion and exclusion criteria

In order to identify articles suitable for the present study, all enrolled articles should meet the including criteria: (1) patients reported in the article were all diagnosed with gold standard (pathological diagnosis); (2) the detection of miR-196a was performed in serum, plasma, tissues or other human body fluids; (3) reported sufficient value related to the expression of miR-196a and prognostic value for overall survival (OS), progression-free survival (PFS), recurrence-free survival (RFS) or disease-free survival (DFS); (4) provided sufficient data to calculate or extract the true positives (TPs), false positives (FPs), false negatives (FNs), and true negatives (TNs).

Additionally, articles that met one of the following terms were removed: (1) non-English and non-Chinese publications; (2) insufficient diagnostic and prognostic data available for meta-analysis.

### Data extraction and checking

Two authors (M.X. and B.P.) independently completed database search, article quality evaluation, data extraction, and uncertain articles were evaluated by the third author (B.H.). The extracted data include author name, publication date, country and region of case, miRNA type, sample type, cancer type, sample size, sensitivity (SEN) and specificity (SPE), cut-off value, HR and 95% CI and follow-up time.

### Statistical analysis

To evaluate the diagnosis value of miR-196a for cancer, the SEN and SPE of all included articles and the corresponding sample content were extracted, and then summary receiver operating characteristic (SROC) curve was drawn based on original data of enrolled studies, and the area under curve (AUC) was used to evaluate the diagnostic value. Chi-square test and *I*^2^ test were applied to assess the heterogeneity across the studies.

In the prognostic meta-analysis, the pooled HR with 95% CI was calculated to evaluate the relationship between the level of miR-196a and the prognosis of cancer patients. Whereas, there were two studies that did not directly present available data [24,25], we obtained the value using the Kaplan–Meier survival curves according to the method reported by Tierney et al. [26]. Similarly, Chi-square test and *I*^2^ test were applied to evaluate the heterogeneity. If *I*^2^ < 50% and *P*>0.05, we use a fixed-effects model, otherwise the random-effects model was applied [27]. To describe the publication bias, funnel plots, Begg’s and Egger’s tests were applied.

All data were carried out with the statistical software STATA (version 13.1) and *P*<0.05 is statistically significant.

### Database analysis

To explore the dysregulated miR-196a expression in cancers, data of the serum samples were obtained from Gene Expression Omnibus (GEO) database. We accomplished a comprehensive analysis of miR-196a expression profiles in GSE113486 and GSE106817. Besides, we also explored the role of miR-196a in cancer prognosis prediction in the ENCORI (http://starbase.sysu.edu.cn/panCancer.php) and Kaplan–Meier Plotter databases (http://kmplot.com/analysis/index.php?p=service), respectively.

## Results

### Eligible studies

After reading full-text of 44 articles meeting the including criteria, 21 of them with insufficient data and unrelated to the diagnosis and prognosis were removed. Finally, a total of 23 articles were enrolled in this meta-analysis (Supplementary Figure S1), of which, 7 studies were related to diagnosis [21,28–33] (Table 1) and 17 studies were related to prognosis [15,16,19,20,24,25,31,34–43] (Table 2), respectively.

**Table 1:** Characteristics and methodology assessment of seven studies included in the diagnosis meta-analysis

First author	Year	City	Ethnicity	Sample type	Cancer type	Case/Control	AUC	SEN (%)	SPE (%)	TP	FP	FN	TN	Cut-off value	MiRNA type
Min [13]	2018	China	Asian	Serum	NSCLC	80/75	0.785	67.86%	77.57%	54	17	26	58	Median	miR-196a-5p
Lu [21]	2015	China	Asian	Plasma	ORC	90/53	0.864	66.70%	96.20%	60	2	30	51	29.9	miR-196a
Wang [24]	2009	America	Caucasian	Plasma	Pancreatic cancer	28/19	0.69	43%	84%	12	3	16	16	NM	miR-196a
Slater1 [22]	2014	Germany	Caucasian	Serum	PanIN2/3	5/10	0.64	100%	60%	5	4	0	6	7.51	miR-196a
Slater2 [22]	2014	Germany	Caucasian	Serum	Sp-FPC	9/10	0.97	90%	89%	8	1	1	9	7.96	miR-196a
Slater3 [22]	2014	Germany	Caucasian	Serum	FPC	10/10	0.99.	90%	100%	9	0	1	10	7.96	miR-196a
Tsai [23]	2016	China	Asian	Plasma	GC	98/126	0.864	69.50%	97.60%	68	3	30	123	1.153	miR-196a
Pan [25]	2020	China	Asian	Serum	Cervical cancer	158/60	0.835	84.2%	80.3%	133	12	25	48	3.84	miR-196a
Liu [20]	2020	China	Asian	Plasma	Pancreatic cancer	40/40	0.865	72.5%	92.5%	29	3	11	37	1.56	miR-196a

Abbreviations: FPC, familial pancreatic cancer; GC, gastric cancer; IPMN, intraductal papillary mucinous neoplasm of the pancreas; ORC, oral cancer; PanIN2/3, pancreatic intrapithelial neoplasia grades 2–3; PanNET, pancreatic neuroendocrine tumor; Sp-FPC, sporadic pancreatic ductal adenocarcinoma.

**Table 2:** The main features of 17 included studies in prognostic meta-analysis

First author	Year	Country	Ethnicity	Sample type	Cancer type	Case	Outcome	HR	(95% CIs)	*P*-value	Cut-off value	MiRNA type
Tsai [23]	2016	China	Asian	Plasma	GC	98	OS	3.057 (M)	1.100–8.495	0.032	Median	miR-196a
Lee [28]	2015	Korea	Asian	Tissues	PanNET	37	OS	16.267 (M)	1.732–153.789	0.015	1.279	miR-196a
Kong [17]	2011	China	Asian	Serum	PDAC	33	OS	2.67 (U)	0.6–11.86	0.007	-5.22	miR-196a
Fu [11]	2018	China	Asian	Tissues	Thyroid cancer	530^*^	OS	5.111 (M)	3.724–7.706	0.008	Median	miR-196a-2
Liu [29]	2013	China	Asian	Tissues	OSCC	95	OS	2.57 (M)	1.20–5.48	0.02	Median	miR-196a
Wang [10]	2019	China	Asian	Tissues	HCC	83	RFS	2.395 (M)	1.207–4.752	0.0125	Median	miR-196a
Niinuma [32]	2012	Japan	Asian	Tissues	GIST	132	OS	9.1 (M)	3.5–23.7	<0.001	1.4	miR-196a
Guan [7]	2015	China	Asian	Tissues	Glioma	63	OS	1.8 (M)	1.2–2.8	0.005	Median	miR-196a
Zhang [35]	2018	China	Asian	Bone marrow	AML	124	OS	1.845 (M)	0.996–3.417	0.052	Median	miR-196a
Fan 1 [26]	2015	China	Asian	Tissues	EOC	146	OS	2.731 (M)	0.804–9.637	0.025	NM	miR-196a
Fan 2 [26]	2015	China	Asian	Tissues	EOC	146	RFS	2.432 (M)	0.638–8.537	0.076	NM	miR-196a
Tang [33]	2018	China	Asian	Tissues	Thyroid cancer	514^*^	OS	2.864 (M)	0.065–4.881	0.147	NM	miR-196a-2
Milevskiy 1 [31]	2019	Australia	Caucasian	Tissues	ER+ breast cancer	-^*^	OS	0.342 (M)	0.1534–0.7623	0.0091	NM	miR-196a
Milevskiy 2 [31]	2019	Australia	Caucasian	Tissues	ER+ breast cancer	-^*^	OS	1.599 (M)	1.0806–2.3652	0.0195	NM	miR-196a
Liu [30]	2015	China	Asian	Serum	Cervical cancer	105	OS	3.510 (M)	1.961–6.874	0.025	NM	miR-196a
Ge 1 [27]	2014	China	Asian	Tissues	CRC	126	OS	4.691 (M)	1.688–10.318	0.001	NM	miR-196a
Ge 2 [27]	2014	China	Asian	Tissues	CRC	126	RFS	4.668 (M)	1.632–10.261	0.001	NM	miR-196a
Zhang 1 [34]	2014	China	Asian	Serum	Osteosarcoma	105	OS	6.28 (M)	1.62–13.39	0.01	4.86	miR-196a
Zhang 2 [34]	2014	China	Asian	Serum	Osteosarcoma	105	RFS	6.95 (M)	1.63–14.82	0.01	4.86	miR-196a
Sun [16]	2012	China	Asian	Tissues	GC	31	OS	2.90 (U)	0.47–17.90	<0.001	Median	miR-196a
Wu 1 [12]	2017	China	Asian	Tissues	Esophageal carcinoma	120	OS	1.985 (M)	1.256–2.961	0.019	Median	miR-196a
Wu 2 [12]	2017	China	Asian	Tissues	Esophageal carcinoma	120	DFS	1.927 (M)	1.343–2.671	0.016	Median	miR-196a

Abbreviations: AML, acute myeloid leukemia; CRC, colorectal cancer; EOC, epithelial ovarian cancer; GC, gastric cancer; GIST, gastrointestinal stromal tumors; OSCC, oral squamous cell carcinoma; PDAC, pancreatic ductal adenocarcinoma; PanNET, pancreatic neuroendocrine tumor; RFS, relapse-free survival.

* Data from TCGA.

To assess the quality of non-randomized researches, the Newcastle–Ottawa Scale (NOS) was applied [44]. We scored each article strictly according to the scoring standard, and those with a score greater than 6 were considered high-quality articles (Table 3).

**Table 3:** Newcastle–Ottawa quality assessments scale

First author	Year	Quality indicators from NOS								Scores
		1	2	3	4	5	6	7	8	
Tsai [23]	2016	+	+	+	-	++	+	+	+	8
Lee [28]	2015	+	+	-	-	++	+	+	+	7
Kong [17]	2011	+	+	+	-	-	+	+	+	6
Fu [11]	2018	+	+	-	-	++	-	+	+	6
Liu [29]	2013	+	+	-	-	++	+	+	+	7
Wang [10]	2019	+	+	-	-	++	+	+	+	7
Niinuma [32]	2012	+	+	-	-	++	+	+	+	7
Guan [7]	2015	+	+	+	-	++	+	+	+	8
Zhang [35]	2018	+	+	-	-	+	+	+	+	6
Fan [26]	2015	+	+	-	-	++	+	+	+	7
Tang [33]	2018	+	+	-	-	++	-	+	+	6
Milevskiy [31]	2019	+	+	-	-	++	-	+	+	6
Liu [30]	2015	+	+	-	-	++	+	+	+	7
Ge [27]	2014	+	+	+	-	++	+	+	+	8
Zhang [34]	2014	+	+	-	-	++	+	+	+	7
Sun [16]	2012	+	+	+	-	-	+	+	+	6
Wu [12]	2017	+	+	-	-	++	+	+	+	7

1. Representativeness of the exposed cohort; 2. Selection of the non-exposed cohort; 3. Ascertainment of exposure; 4. Outcome of interest not present at the start of study; 5. Control for important factor or additional factor; 6. Assessment of outcome; 7. Follow-up long enough for outcomes to occur; 8. Adequacy of follow-up of cohorts.

### Diagnostic meta-analysis

#### Study characteristics

Seven articles reported the role of miR-196a as a biomarker in cancer diagnosis (Table 1), and all the samples of these studies were collected as serum and plasma. For ethnicity, there were two and five studies based on European and Asian populations, respectively. The quantitative real-time polymerase chain reaction (qRT-PCR) was used by all studies to detect miRNA expression.

#### Expression of miR-196a and diagnosis

**Figure 1:**
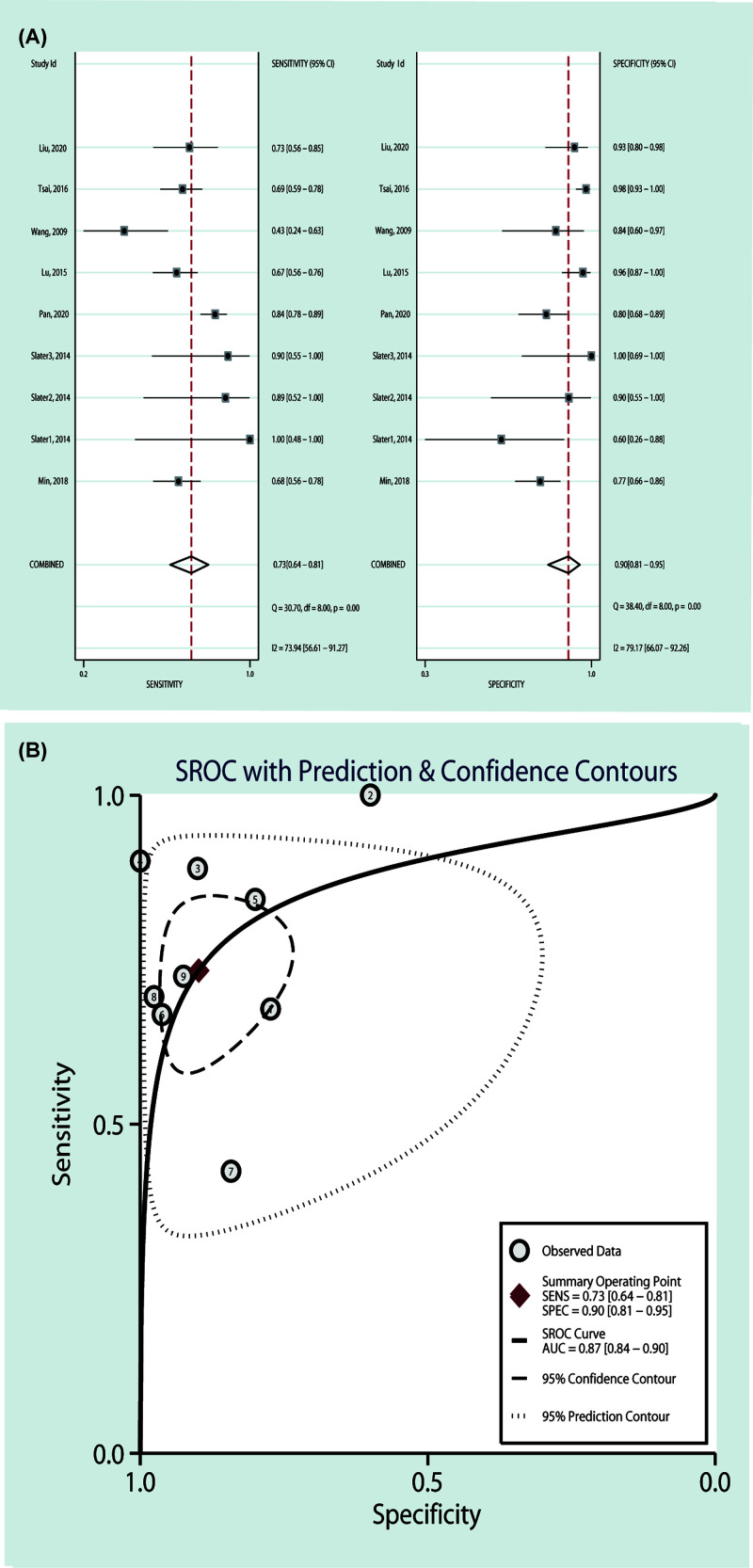
The association of miR-196a expression level and cancer diagnosis (**A**) Forest plots and (**B**) SROC curve revealed that miR-196a is a valuable diagnostic biomarker for cancers.

In order to assess the diagnostic value of miR-196a for cancer, the pooled SEN and SPE were calculated, and forest plots were also drawn (Figure 1). The pooled AUC (AUC = 0.87, 95% CI: 0.84–0.90; SEN = 0.73, 95% CI: 0.64–0.81; SPE = 0.90, 95% CI: 0.81–0.95) (Figure 1, Table 4) indicated that miR-196a is a valuable diagnostic biomarker for cancers.

**Table 4:** Results of diagnostic meta-analysis

Variables	Subgroup	Case/Control	Pooled results						
			AUC (95% CI)	SEN (95% CI)	*I*^2^ (%)	*P*	SPE (95% CI)	*I*^2^ (%)	*P*
Serum and Plasma	-	518/403	0.87 (0.84–0.90)	0.73 (0.64–0.81)	73.94	0.00	0.90 (0.81–0.95)	79.17	0.00
Serum and Plasma	Asian	466/354	0.86 (0.83–0.89)	0.73 (0.66–0.79)	70.87	0.01	0.92 (0.81–0.97)	86.60	0.00
Serum and Plasma	Caucasian	52/49	0.90 (0.87–0.92)	0.85 (0.44–0.98)	83.22	0.00	0.84 (0.64–0.94)	67.12	0.03
Serum and Plasma	Sample size < 100	92/89	0.91 (0.88–0.93)	0.80 (0.50–0.94)	75.86	0.00	0.87 (0.74–0.94)	63.93	0.03
Serum and Plasma	Sample size > 100	426/314	0.84 (0.80–0.87)	0.73 (0.64–0.80)	78.13	0.00	0.91 (0.77–0.97)	89.45	0.00

**Figure 2:**
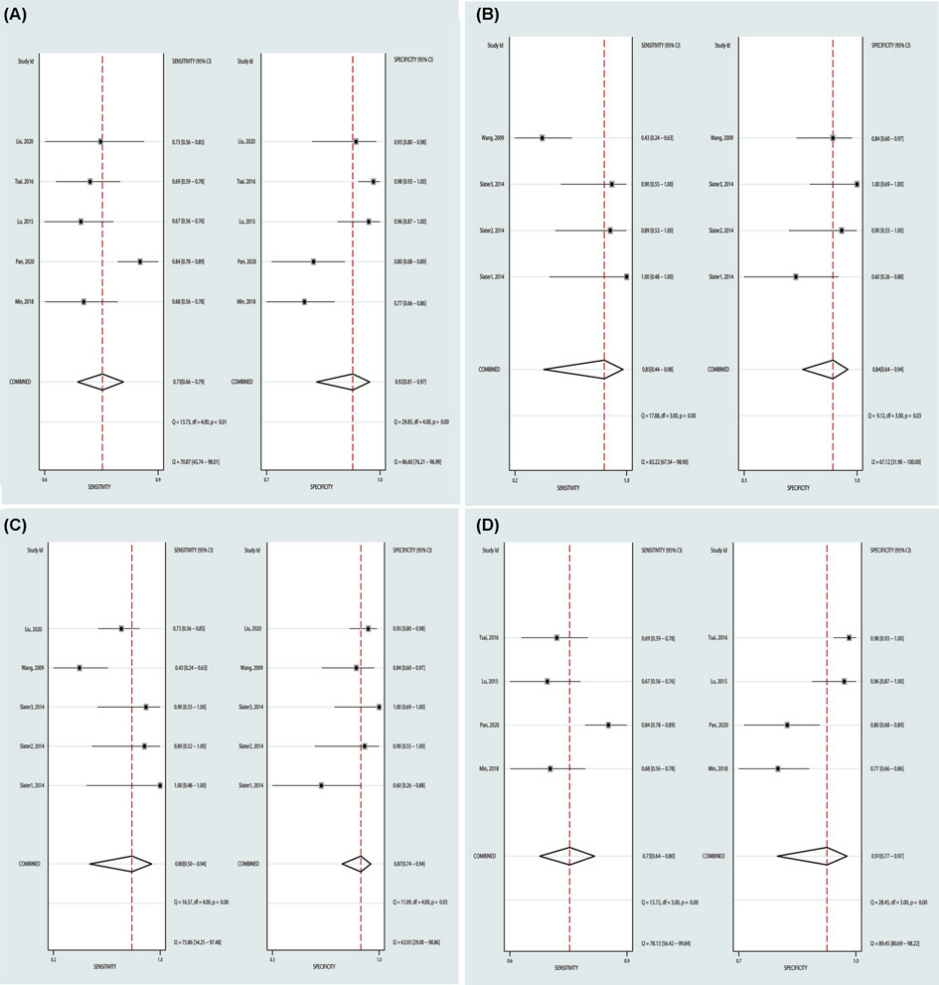
Subgroup analyses between miR-196a expression levels and cancer diagnosis (**A**) Asian subgroup, (**B**) Caucasian subgroup, (**C**) Sample size < 100 subgroup and (**D**) Sample size > 100 subgroup results all showed that miR-196a is a valuable diagnostic biomarker for cancers.

In order to assess the diagnostic value of miR-196a for cancer among subgroups, we separated the studies according to sample size (more than 100 or not) and ethnicities (Asian or Caucasian), and subgroup analysis revealed that the results of subgroup stratified by sample size (Sample size < 100: AUC = 0.91, 95% CI: 0.88–0.93; SEN = 0.80, 95% CI: 0.50–0.94; SPE = 0.87, 95% CI: 0.74–0.94; Sample size > 100: AUC = 0.84, 95% CI: 0.80–0.87; SEN = 0.73, 95% CI: 0.64–0.80; SPE = 0.91, 95% CI: 0.77–0.97) or ethnicity (Asian: AUC = 0.86, 95% CI: 0.83–0.89; SEN = 0.73, 95% CI: 0.66–0.79; SPE = 0.92, 95% CI: 0.81–0.97; Caucasian: AUC = 0.90, 95% CI: 0.87–0.92; SEN = 0.85, 95% CI: 0.44–0.98; SPE = 0.84, 95% CI: 0.64–0.94); all had significant differences, which were consistent with overall pooled results (Figure 2, Table 4).

### Prognostic meta-analysis

#### Study characteristics and quality assessment

In order to explore the relationship between miR-196a expression and survival of cancer patients, a total of 17 articles were enrolled in this meta-analysis. Of enrolled studies, a total of 12 studies were conducted with tumor tissue, 4 articles involving serum or plasma, and only 1 article based on bone marrow samples [43]. In addition, all the 17 articles were based on Asians except 1 based on Caucasians [39]. All detection methods of included studies were based on qRT-PCR, as shown in Table 2.

#### Expression of miR-196a and prognosis

**Figure 3:**
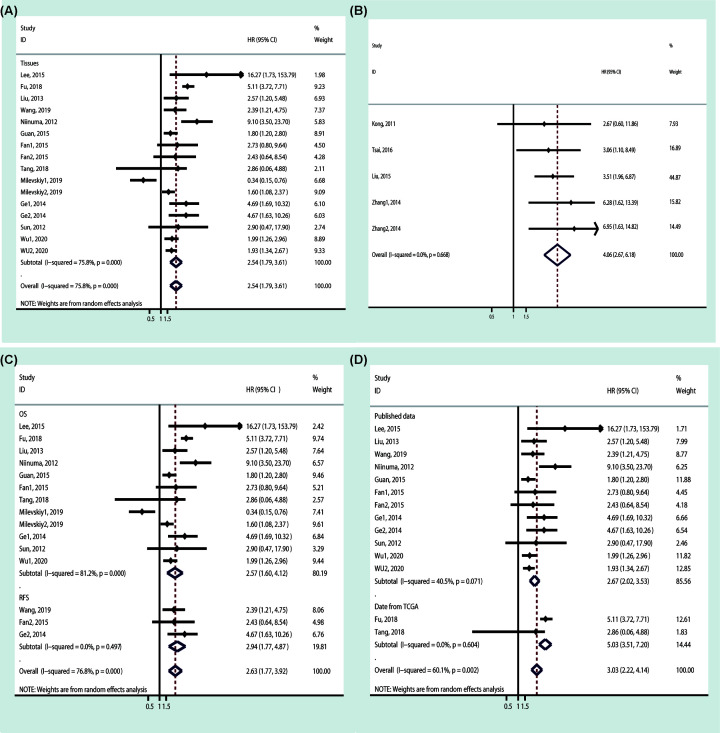
The association of miR-196a expression level with survival in cancer patients with different cancer type (**A**) Tissues sample type and (**B**) Serum and plasma sample type pooled results revealed that miR-196a is a valuable prognostic biomarker for cancers. **Subgroup analysis results with tissue sample type revealed that miR-196a is a valuable prognostic biomarker for cancers**: (**C**) survival data subgroup and (**D**) data resources subgroup.

The pooled results of all 12 studies conducted with tumor tissue showed that the increased expression of miR-196a was an unfavorable survival prognosis biomarker (high expression *vs.* low expression: HR = 2.54, 95% CI: 1.79–3.61). The similar result was also observed in those studies conducted with serum or plasma (high expression *vs*. low expression: HR = 4.06, 95% CI: 2.67–6.18) (Figure 3, Table 5).

**Table 5:** Results of prognostic meta-analysis

Variables	Subgroup	Pooled HR (95% CI)	*I^2^*(%)	*P*
Serum and Plasma	-	4.06 (2.67–6.18)	0	0.668
Tissues	-	2.54 (1.79–3.61)	75.8	0.000
Tissues	OS	2.57 (1.60–4.12)	81.2	0.000
Tissues	RFS	2.94 (1.77–4.87)	0	0.497
Tissues	Published data	2.67 (2.02–3.53)	40.5	0.071
Tissues	Data from TCGA	5.03 (3.51–7.20)	0	0.604

To assess the pooled result further, we performed subgroup analysis according to survival data (OS or RFS) and the data resources (published data or TCGA data), and the result showed that the pooled results of all subgroups (OS: HR = 2.57, 95% CI: 1.60–4.12; RFS: HR = 2.94, 95% CI: 1.77–4.87; published data: HR = 2.67, 95% CI: 2.02–3.53; data from TCGA: HR = 5.03, 95% CI: 3.51–7.20) were similar to the overall pooled result (Figure 3, Table 5).

#### Heterogeneity and sensitivity analyses

**Figure 4:**
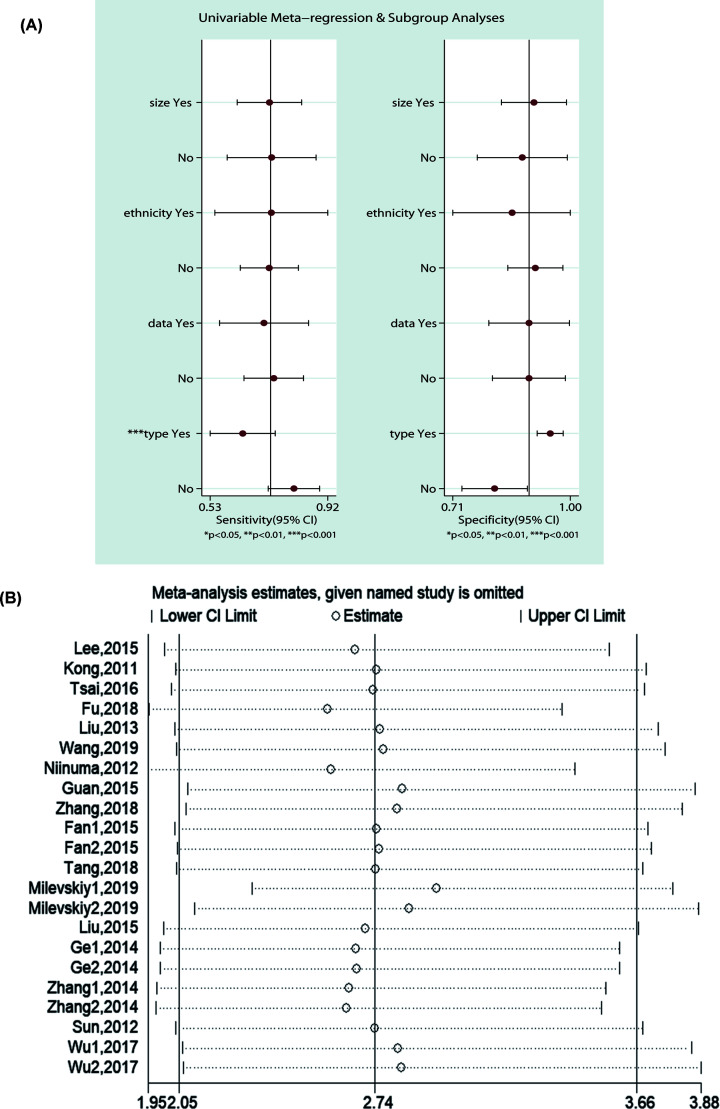
Herterogeneity analysis to detect the diagnostic and prognostic heterogeneity origin (**A**) Meta-regression showed that heterogeneity may come from sample type. (**B**) Sensitivity analyses; no single study deletion changed the results.

For the meta-analysis of diagnosis, among the studies conducted with serum or plasma, there was a significant heterogeneity across the enrolled studies (*P_Heterogeneity_*<0.001, *I*^2^ = 73.9%) and subgroup of sample size (*n*<100: *P_Heterogeneity_*<0.001, *I*^2^ = 75.86%; *n*<100: *P_Heterogeneity_*<0.001, *I*^2^ = 78.13%) and ethnicity (Asian: *P_Heterogeneity_*=0.01, *I*^2^ = 70.87%; Caucasian: *P_Heterogeneity_*=0.00, *I*^2^ = 83.22%). Therefore, a meta-regression was conducted based on sample size, ethnicity and year of publication. The results suggested that heterogeneity was mainly derived from sample type (*P*<0.001) (Figure 4).

For prognosis analysis, there was no significant heterogeneity among the studies involving serum or plasma. Whereas, there was a significant heterogeneity across the studies based on the sample type of tumor tissue (*P_Heterogeneity_*<0.001, *I*^2^ = 75.80%) and subgroup of studies with OS (*P_Heterogeneity_*<0.001, *I*^2^ = 81.20%), which may be due to the difference of data resources in that the heterogeneity was decreased (HR = 2.67, 95% CI: 2.02–3.53, *P_Heterogeneity_*=0.071, *I*^2^ = 40.5%) when three studies come from the database were removed [19,39,41]. Additionally, to assess the stability of the pooled result, a sensitivity analysis was conducted by omitting each study and the result revealed that no single study deletion changed the significance of the pooled result (Figure 4).

#### Publication bias

**Figure 5:**
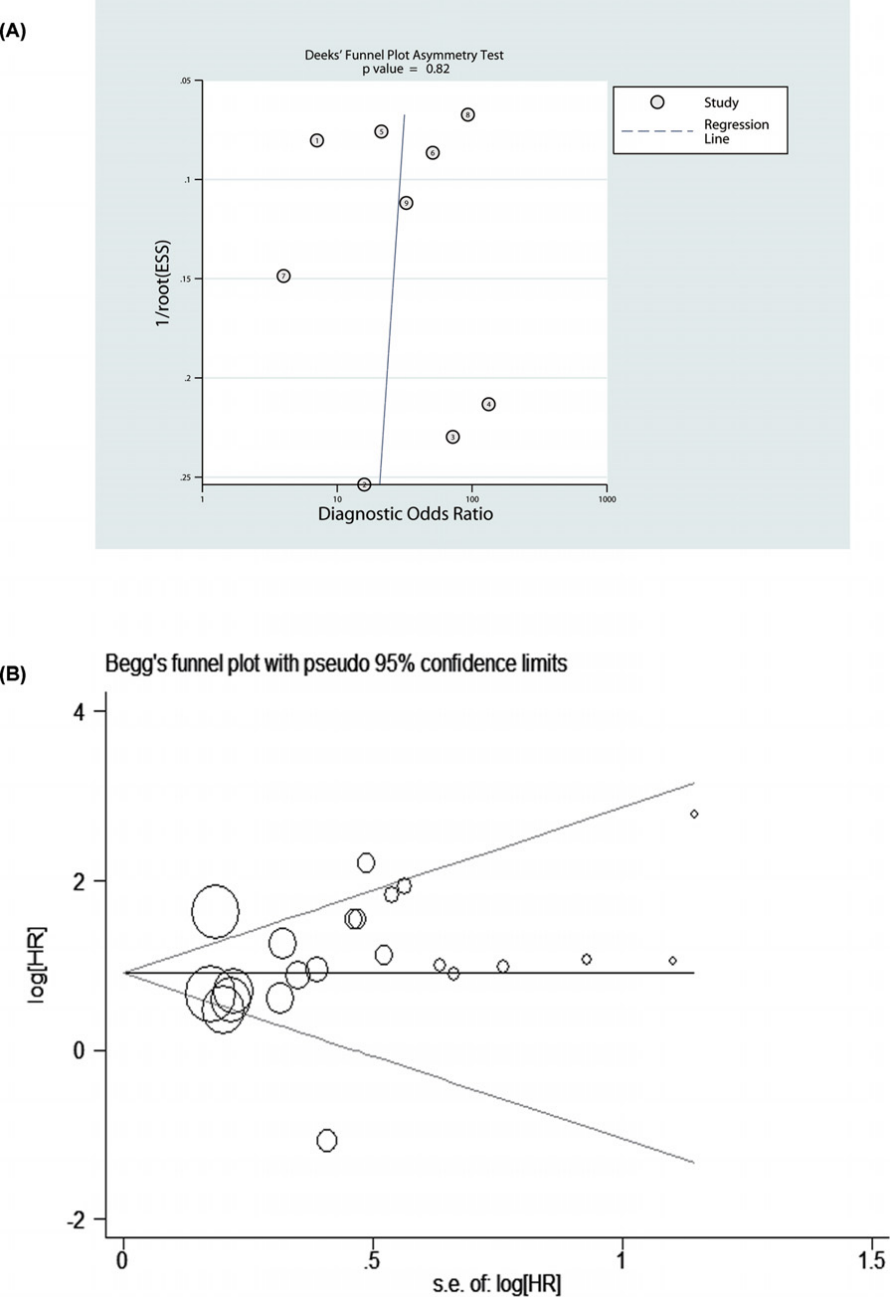
Publication bias analysis (**A**) Diagnostic publication bias analysis and (**B**) prognostic publication bias analysis revealed that there was no publication bias.

To test the publication bias of the studies based on diagnosis, Deeks’ funnel plot asymmetry test was used. The funnel plots of the studies related diagnosis were symmetrical, indicating no publication bias of these studies was presented (*t* = −0.24, *P*=0.816). Additionally, the Egger’s and Begg’s tests were performed for the studies related to prognosis, the similar results was observed (*t* = 1.16, *P*=0.260), shown in Figure 5.

#### Diagnostic and prognostic analyses based on the database

In order to verify the diagnostic role of miR-196a in serum of patients with cancer, we searched two datasets in the GEO database (GSE113486 and GSE106817) containing expression of miR-196a in breast cancer, pancreatic cancer patients and corresponding normal controls, and results showed that the AUC of miR-196a-3p (AUC = 0.77, 95% CI: 0.74–0.79) and -5p (AUC = 0.71, 95% CI: 0.66–0.75) showed favorable diagnostic values for breast cancer and pancreatic cancer (miR-196a-3p: AUC = 0.80, 95% CI: 0.73–0.87; miR-196a-5p: AUC = 0.61, 95% CI: 0.51–0.71), respectively, which were consistent with the pooled results of the present study.

**Figure 6:**
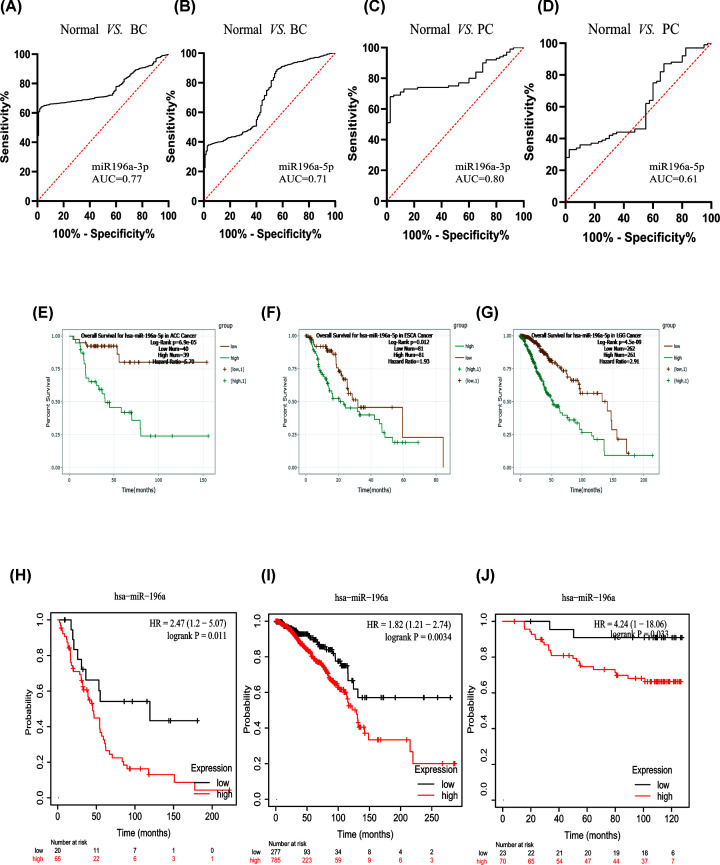
Results from database showed that miR-196a is a valuable diagnostic and prognostic biomarker for cancers ROC curve of (**A**) miR-196a-3p in breast cancer, (**B**) miR-196a-5p in breast cancer, (**C**) miR-196a-3p in pancreatic cancer, (**D**) miR-196a-5p in pancreatic cancer. K–M plotter of miR-196a in (**E**) adrenocortical carcinoma, (**F**) esophageal carcinoma, (**G**) brain lower grade glioma. K–M plotter of miR-196a in breast cancer of (**H**) GSE40267, (**I**) TCGA, (**J**) GSE19783.

In order to verify the prognosis of miR-196a for cancer, we searched in the online databases ENCORI, which contains survival and differential expression analyses of miRNAs, lncRNAs, pseudogenes and mRNAs and in Kaplan–Meier Plotter database, which includes the effect of mRNA, miRNA, protein on survival in 21 cancer types. As shown in Figure 6, the prognostic HR values of miR-196a-5p in patients with adrenocortical carcinoma, esophageal carcinoma, and brain lower grade glioma were 5.70 (*P*=6.9e-5), 1.93 (*P*=0.012), 2.91 (*P*=4.5e-9), respectively. In addition, the results of Kaplan–Meier Plotter database showed that high expression of miR-196a predicted unfavorable OS of breast cancer patients (GSE40267: HR = 2.47, 95% CI: 1.2–5.07, *P*=0.011; TCGA: HR = 1.82, 95% CI: 1.21–2.74, *P*=0.0034; GSE19783: HR = 4.24, 95% CI: 1–18.06, *P*=0.033). Therefore, all the results from databases supported the pooled results based on published data.

## Discussion

In this meta-analysis, a total of 23 articles were included to explore the role of miR-196a in cancer diagnosis and prognosis. The pooled results showed that the expression of miR-196a could be used as a diagnosis and prognosis biomarker for cancers.

For diagnosis meta-analysis, in the present study, a total of seven diagnosis-related articles were included, the overall and subgroups pooled result showed that miR-196a could be used as a diagnostic marker for cancer. In fact, the oncogene role of miR-196a in cancer has been reported by studies, and it combined with other miRNAs can improve the efficiency of cancer diagnosis. Such as miR-196a and miR-148a could act as candidate biomarkers for early gastric cancer (GC) diagnosis [45], the combination of miR-10a-5p and miR-196a-5p can serve as non-invasive biomarkers for NSCLC [21], and miR-196a combined with miR-1202 could serve as biomarkers for evaluating the effectiveness of endometrial cancer treatment [46]. In addition, results from databases were consistent to the pooled results, indicating miR-196a has promising clinical application in cancer diagnosis.

The mechanisms of overexpression of miR-196a in cancer have been illustrated by previous studies. In breast cancer, miR-196a could be transcriptionally regulated by the binding of ERα to its promoter region and DNA methylation within the HOXC locus negatively related with the expression of miR-196a, supporting the report that miR-196a could be regulated in a repressive epigenetic modification [5]. Moreover, a time delay was found in the precursor *MIR196A2* gene into mature MIR196A processing, suggesting the overexpression of miR-196a was regulated post-transcriptionally [39].

In this meta-analysis, a significant heterogeneity among enrolled diagnosis related studies was presented in the overall and subgroup results, which was attributed to the types of the sample, suggesting that the level of miRNAs may be affected according the sample type. Actually, the difference of miRNAs level in serum and plasma has been reported previously, which may be attributed to the some detectable miRNAs were from platelets [47].

Regarding the role of miR-196a in the prognosis of cancer, the overall and subgroups pooled results showed that miR-196a could be used as a prognostic marker for cancer. Actually, miR-196, regarding as an oncogene, has been investigated with several biological function-related tumor progression. High expression of miR-196a was associated with shorter OS of GC patients, which may be attributed to the down-regulation of its targeted gene *p27kip1* [24]. Moreover, miR-196a promoted tumor progression by down-regulation of SPRR2C, S100A9 and KRT5 [48]. Additionally, in colorectal cancer (CRC), miR-196 could lead to metastasis by inhibiting HoxB8, and it can also decrease the sensitivity of cancer cells to chemotherapy with FOLFOX4, resulting in unfavorable prognosis [49], supporting it is a favorable prognostic biomarker.

For the meta-analysis of prognosis, the pooled results of this article indicated that high expression of miR-196a predicted the poor prognosis of cancer patients. Whereas, a significant heterogeneity was presented among studies, which could be eliminated by removing three studies coming from the database that two were thyroid cancer data from TCGA database and one breast cancer data from GEO database. Specifically, in the breast cancer study, the opposite HR of miR-196 to survival of patients was reported for patients with the ER+ pre-menopausal (HR = 0.342, 95% CI: 0.1534–0.7623) and ER+ post-menopausal (HR = 1.599, 95% CI: 1.0806–2.3652), which may be a source of heterogeneity. More important, the original data of these three studies were based on high-throughput platform, which was different with other studies based on qRT-PCR, may contribute to the heterogeneity. In short, the pooled results of published data or results of databases all supported that high expression of miR-196a predicted the poor prognosis of cancer patients.

Admittedly, there have been previous meta-analysis articles regarding the role of miR-196a in cancer diagnosis and prognosis. For example, the prognostic value of miR-196a was assessed in Asian cancer patients [50]. Compared with this article, the novelty of the present study was as follows: (1) we included more recent studies, regarding European population, Asian population and more cancer type, indicating the conclusion of the present study was robust; (2) we also retrieved the data of related databases (GEO, K–M Plotter, ENCORI) to confirm the pooled results of published data, which was consistent each other, indicating our result was based on a larger size of sample; (3) we further proved the feasibility of miR-196a as a cancer diagnostic biomarker in serum or plasma based on published data and data of databases, indicating our study was relatively more comprehensive. In addition, compared with the study regarding the polymorphism locates at the coding region of miR-196a [51], our study discussed the expression of miR-196a, and our previous study has reported the association between the miR-196a polymorphism and cancer risk [10].

Although, the result of meta-analysis was objective and robust, some limitations of this article should be addressed. First, the HR and corresponding 95% CIs of two articles were extracted from survival curves, which may be not objective enough and have an impact on the final results. Second, all the studies published in English or Chinese were included, which may lead to the language bias. Third, the results of this meta-analysis lack experiments to confirm, which should be validated by future study.

## Conclusion

In short, our study concluded that miR-196a can be used as a diagnostic and prognostic marker for cancers.

## Supplementary Material

Supplementary Figure S1

## Data Availability

The data are available from the corresponding author (B.H.) upon reasonable request. All data generated or analyzed during the present study are included in this published article.

## Abbreviations

AUC: area under curve
CI: confidence interval
FN: false negative
FOXO1: forkhead box transcription factor O1
FP: false positive
FPC: familial pancreatic cancer
GC: gastric cancer
GEO: Gene Expression Omnibus
HCC: hepatocellular carcinoma
HR: hazard ratio
miRNA: microRNA
mRNA: messenger RNA
NSCLC: non-small cell lung cancer
OS: overall survival
qRT-PCR: quantitative real-time polymerase chain reaction
RFS: recurrence/relapse-free survival
SEN: sensitivity
SPE: specificity
SPRED-1: sprouty-related, EVH1 domain-containing protein 1
TCGA: the cancer genome atlas
