# Supplementary figures and images for: The diagnostic and prognostic values of microRNA-196a in cancer
Source: Biosci Rep. 2021 Jan 7;41(1):BSR20203559. doi: 10.1042/BSR20203559 (PMC7791550; doi:10.1042/BSR20203559)

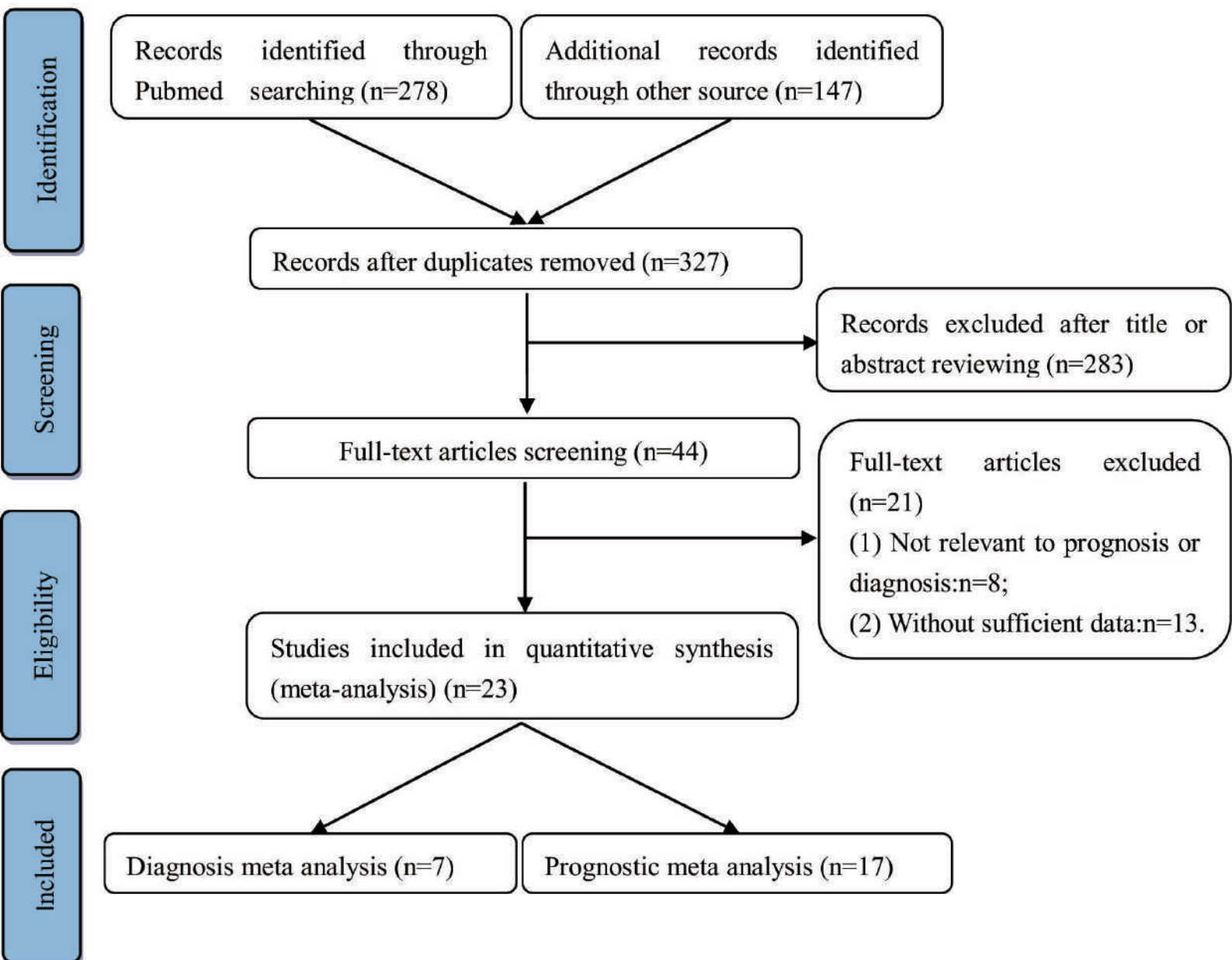

Supplement: Supplementary Figure S1 [file BSR-2020-3559_supp.pdf]
